# Role of Metformin and AKT Axis Modulation in the Reversion of Hypoxia Induced TMZ-Resistance in Glioma Cells

**DOI:** 10.3389/fonc.2019.00463

**Published:** 2019-05-31

**Authors:** Alessia Lo Dico, Silvia Valtorta, Luisa Ottobrini, Rosa Maria Moresco

**Affiliations:** ^1^Department of Pathophysiology and Transplantation (DEPT), University of Milan, Milan, Italy; ^2^Institute of Molecular Bioimaging and Physiology (IBFM), National Research Council (CNR), Segrate, Italy; ^3^Tecnomed Foundation, Medicine and Surgery Department, University of Milano- Bicocca, Monza, Italy; ^4^Experimental Imaging Center, IRCCS San Raffaele Scientific Institute, Milan, Italy

**Keywords:** MET, TMZ responsiveness, hypoxia resistance, AKT pathway, HIF-1α biomarker

## Abstract

Hypoxia is a key driver of tumor adaptation promoting tumor progression and resistance to therapy. Hypoxia related pathways might represent attractive targets for the treatment of Glioblastoma Multiforme (GBM), that up to date is characterized by a poor prognosis. Primary aim of this study was to investigate the role of hypoxia and hypoxia-related modifications in the effect of temozolomide (TMZ) given alone or in association with the antidiabetic agent Metformin (MET) or the PI3K/mTOR blocker, BEZ235. The study was conducted in the TMZ responsive U251 and resistant T98 GBM cells. Our results showed that during hypoxia, TMZ plus MET reduced viability of U251 cells affecting also CD133 and CD90 expressing cells. This effect was associated with a reduction of HIF-1α activity, VEGF release and AKT activation. In T98 TMZ-resistant cells, TMZ plus MET exerted similar effects on HIF-1α. However, in this cell line, TMZ plus MET failed to reduce CD133 positive cells and AKT phosphorylation. Nevertheless, the administration of the dual PI3K/mTOR inhibitor BEZ235 potentiated the effect of TMZ plus MET on cell viability, inducing a pro-apoptotic phenotype during hypoxic condition also in T98 cells, suggesting the block of the PI3K/AKT/mTOR pathway as a complementary target to further overcome GBM resistance during hypoxia. In conclusion, we proposed TMZ plus MET as suitable treatment to revert TMZ-resistance also during hypoxia, an effect potentiated by the inhibition of PI3K/mTOR axis.

## Introduction

In Glioblastoma Multiforme (GBM), the “international gold standard” therapy includes surgery, radiotherapy, and chemotherapy with Temozolomide (TMZ) (1), but the prognosis remains poor. The glycolytic switch and hypoxia-induced factors influence tumor progression and resistance to therapy (2, 3). Indeed, hypoxia and hypoxia-related factors are associated with pseudopalisading necrotic regions that protect stem cells niche, contributing to the aggressive profile of these tumors (3, 4). Hypoxia effect on tumor malignancy involves hypoxia-inducible factors (5), stem population (6), negative modulation of apoptotic axis (7) and molecular pathways related to regulation of cell metabolism including PI3K/AKT/mTOR pathway (8).

Metformin (MET), a modulator of cell metabolism acting on 5′ adenosine monophosphate-activated protein kinase (AMPK) and mitochondrial respiration, potentiates the activity of TMZ in glioblastoma models (9, 10). Despite the various hypothesis suggested including ours (11–13), the precise mechanism of action of MET remains elusive particularly regarding its activity under hypoxic condition.

As previously stated, hypoxia influences metabolic pathway related to the control of cell metabolism including PI3K/AKT/mTOR favoring tumor proliferation and resistance (14). However, PI3K/AKT signaling is activated by loss of function mutations or reductions of mitochondrial PTEN (15), a molecular feature common in GBM (more than 40%). For this reason, in presence of activating mutations, PI3K/AKT/mTOR pathway influences GBM cell growth also during normoxic conditions. Indeed, administration of the dual PI3K and mTOR inhibitor, BEZ235, is able to reduce GBM chemoresistance, leading G1 cell cycle arrest and downregulation of vascular endothelial growth factor (VEGF) (16–18).

For the reasons above, in this study we initially investigated the role of hypoxia and hypoxia-related modifications on the effect of Temozolomide given alone or in association with drug targeting cell metabolism such as Metformin.

In detail, we tested the role of hypoxia on the add-on effect of MET on TMZ (COMBO), on cell viability, HIF-1α activity, AKT phosphorylation and VEGF release. The study was conducted in two different GBM cell lines, i.e., the TMZ-responsive U251 and the -resistant T98, both carrying mutation in PTEN, previously evaluated under normoxic condition. Subsequently, in order to test the specific role of PI3K/AKT/mTOR pathway, the effect of COMBO was evaluated also in presence of the dual PI3K/mTOR inhibitor BEZ235.

## Materials and Methods

### Cell Culture and Reagents

U251-HRE and T98 human glioma cells (both TP53 and PTEN mutated) were routinely maintained in RPMI supplemented with 10% heat-inactivated fetal bovine serum, penicillin, and streptomycin (50 IU/ml), 2 mM glutamine (Euroclone, UK), 25 mM Hepes Buffer. Cells have been maintained in a humidified atmosphere of 5% of CO_2_ at 37°C. To perform hypoxia experiments, both cell lines were seeded at 10,000 cells/cm^2^ and incubated in a “Hypoxic Chamber” containing 1% O_2_ gas mixture with or without 25 μM TMZ (dissolved in 90% PBS and 10% Dimethyl sulfoxide, DMSO), 10 mM MET (dissolved in 100% PBS) (both Sigma- Aldrich, St. Louis, MO, USA) for 24, 48, and 72 h for cell viability and for 48 h for all other tests and 1 μM BEZ235 (dissolved in 100% DMSO) for 48 h (SelleckChem, Houston, TX, USA). Treatment with TMZ plus Met was defined as COMBO. Cell viability was evaluated using the Trypan blue exclusion test.

### HIF-1α Transcription Activation Assay

Nuclear extracts were prepared by using Nuclear Extract Kit from Vincibiochem (#40010 - Activemotif, CA, USA) according to the manufacturer's instructions. The transcriptional activity of HIF-1α was assayed by an ELISA-based kit [#47096- TransAM Kit, Vinci-Biochem, Vinci (FI), Italy] according to the manufacturer's instructions. Nuclear extract samples were added to the coated plate and analyzed with GloMax-Multi Detection System (Promega, Milan, Italy). Data are expressed as HIF-1alpha activity (ABS OD 450 nm).

The evaluation of HIF-1 target gene was performed by measuring the concentration of VEGF in glioma cell-conditioned medium quantified using an ELISA kit (VEGF Human ELISA KitNovex®, Cat. No. KHG011, Life Technologies, Monza, Italy) in accordance with the manufacturer's protocol. The medium was collected under normoxic and hypoxic conditions. The data are expressed in pg/mL.

### RNA Extraction and Real-Time PCR

RNA was extracted using the commercially available illustraRNAspin Mini Isolation Kit (GE Healthcare, Italy), according to manufacturer's instructions. Total RNA was reverse-transcribed to cDNA using the High Capacity cDNA Reverse Transcription Kit (Applied Biosystem, USA). Real-time PCR was performed in duplicate for each data point, and the oligonucleotides used were as in Table 1. Changes in the target mRNA relative to housekeeping (β-actin) were determined with the ddct Method.

**Table 1:** Primers used for Real-time PCR.

**Gene**	**Forward**	**Reverse**
β-actin	TCAAGATCATTGCTCCTCCTG	CCAGAGGCGTACAGGGATAG
Bax	ATG GAC GGG TCC GGG GAG	ATCCAGCCCAACAGCCGC
Bad	CCCAGAGTTTGAGCCGAGTG	CCCATCCCTTCGTCGTCCT
Bcl-2	GATTGTGGCCTTCTTTGAG	CAAACTGAGCAGAGTCTTC

### FACS Analysis

One hundred thousand cells were washed in PBS and incubated with 0.5 μg of: CD90-FITC, human (clone: DG3)-FITC, human 130-095-403; Monoclonal CD133/2 (293C3)-FITC, human 130-090-853; CD44-FITC, mouse (clone: IM7.8.1) 130-102-511; CD73-PE, human (clone: AD2)-FITC, human 130-095-182 (MiltenyiBiotec). Viable cells were gated by forward and side scatters and analyzed on 100,000 acquired events for each sample (PartecCyFlow Space using the PartecFloMax® software).

### Bioluminescent Assay

For cell transfection, the glioma cell lines were seeded at 10,000 cells/cm^2^ and transfected with pHRE-luciferase plasmid (kindly provided by Dr. G. Melillo, National Cancer Institute, Frederick, MD, USA) that expresses the luciferase reporter gene under the control of three copies of the Hypoxia-Response Element (HRE) sequence (pGL2Tk-HRE) (19). The cells were analyzed using the GloMax-Multi Detection System (Promega, Milan, Italy). Protein content was measured by means of a Bradford assay, and the bioluminescence signal was normalized to milligrams of protein and expressed as relative luminescence units (RLU = luciferase counts per milligram of proteins). The cells were also tested for Caspase-3 activity by Caspase-Glo® Assay (Promega). Briefly, 10,000 cells/cm^2^ were treated, as previously described, and the relative luminescence units at the end of the treatment was detected using a plate reader (Glomax) and after protein normalization, data were expressed as RLU (Relative Luminescence units).

### ELISA AKT

Ten thousand cells/cm^2^ were treated as previously described and, at the end of the treatments, U251 and T98 cell pellets were lysed for 30 min. After protein quantification, lysates were used to assess pAKT [pS473]. A monoclonal antibody specific for AKT has been coated onto the wells of the microtiter strips provided (AKT [pS473] Phospho-ELISA Kit, HumanCatalog number: KHO0111 ThermoFisher). The absorbance was read at 450 nm and after plot on graph paper data were expressed as units of pAKT/ml.

### Statistical Analysis

*in vitro* experiments were repeated three times, giving reproducible results. Data are presented as mean values ± standard deviation (SD) of three independent experiments. For statistical analysis *t*-test or one- or two-way analysis of variance (ANOVA), followed by Dunnett's or Bonferroni's multiple comparison tests, were performed using Prism 4 (GraphPadSoftwareInc., CA, USA).

## Results

### COMBO Reduces the Hypoxia-Dependent Increase of Cell Viability and HIF-1α Activation, Also in T98 Cells

As we have previously shown in a dose-response assay (12), 25 μM TMZ is the correct dose to distinguish the drug sensitivity of U251 and T98 cell lines. Here, we evaluated the effect of 25 μM TMZ and 10 mM MET in combination (COMBO) on HIF-1α transcriptional factor in the two cell lines both in normoxia and in hypoxia. To this aim, both cell lines were incubated for 6 h in a hypoxia chamber and then were analyzed by using an ELISA-based assay for HIF-1 nuclear form.

**Figure 1:**
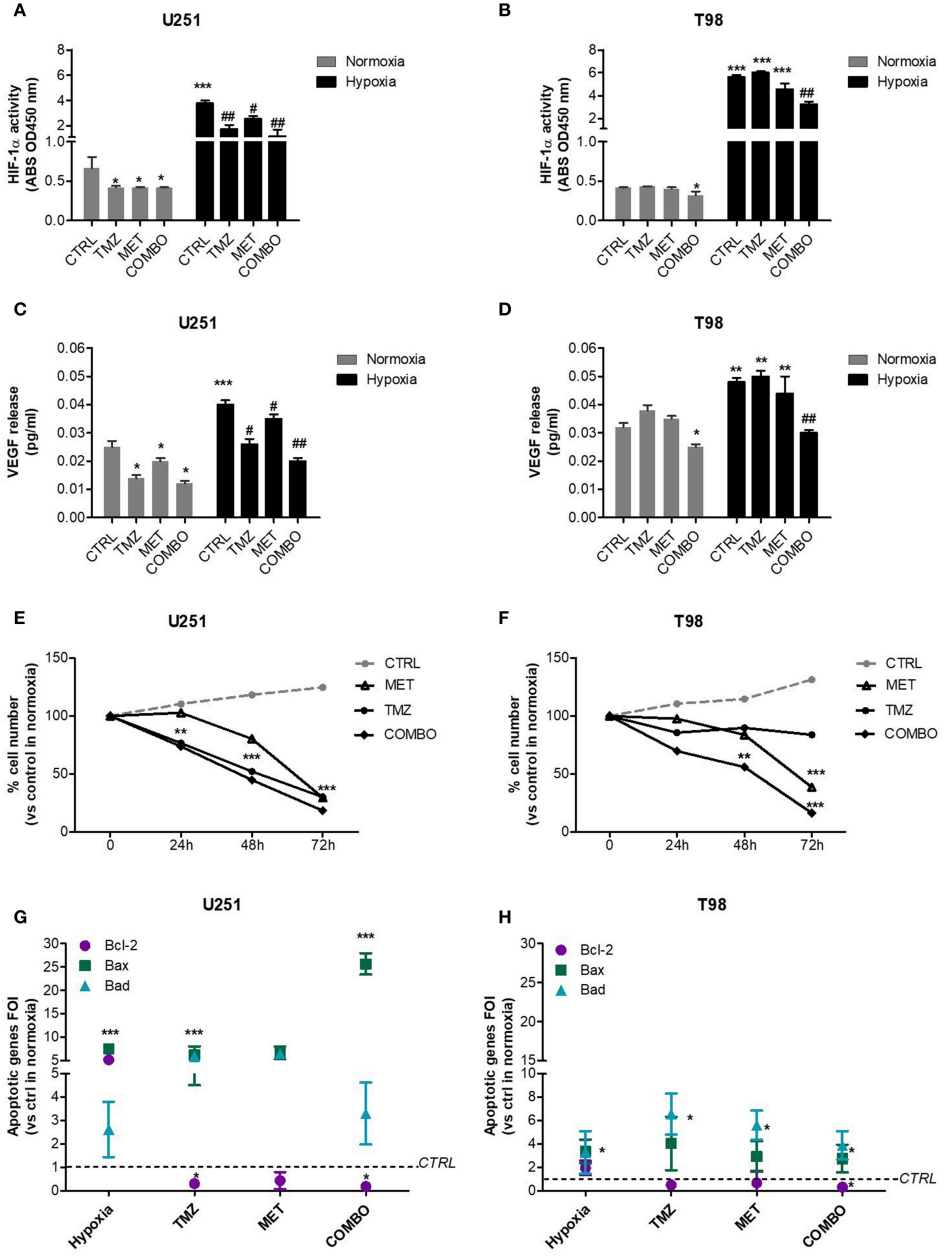
HIF-1α activity modulation and consequences on the apoptotic pattern. ELISA-based HIF-1α activity quantification after 48 h of 25 μM TMZ, 10 mM MET, or COMBO treatment under normoxic and hypoxic conditions in U251 **(A)** and T98 **(B)** cells. The data are expressed as absorbance at 450 nm. *p < 0.05, ***p < 0.001 vs. control under normoxic conditions; #*p* < 0.05, ##*p* < 0.001 vs. control under hypoxic conditions. The evaluation of HIF-1 target gene, VEGF, was performed by the ELISA of the VEGF released by U251 **(C)** and T98 **(D)** glioma cells in cell medium after 25 μM TMZ, 10 mM MET or COMBO treatment. *p < 0.05, ***p < 0.001 vs. control under normoxic conditions; #*p* < 0.05, ##*p* < 0.001 vs. control under hypoxic conditions. Evaluation of time-response viability of responsive and resistant cells after TMZ or COMBO. Cell viability was assessed by means of a Trypan blue exclusion test and expressed as the percentage of viable cells after 24, 48, or 72 h of treatment under hypoxic condition in U251 **(E)** and T98 **(F)** cells. **p < 0.01; ***p < 0.001 vs. control cells. The induction of pro-apoptotic (Bad and Bax) and anti-apoptotic genes (Bcl-2) was analyzed by means of real-time PCR in glioma cells treated with TMZ under hypoxic condition **(G,H)**. The data were normalized to β-actin, and the ΔΔct values were expressed as the ratio between the mean values in the responsive and resistant cells [Fold of Induction (FOI)]. *p < 0.05; ***p < 0.001 treated vs. control cells. Mean values ± SD of three independent experiments.

In both cell lines, we observed a dramatic increase in the transcriptional activity of HIF-1α induced by hypoxia. In U251 cells, all single and combined treatments were able to significantly reduce HIF-1α activity, also in hypoxia (Figure 1A). Otherwise, in T98 TMZ-resistant cells, only COMBO significantly reduced the activity of HIF-1α compared to controls (Figure 1B). However, in both cell lines, neither single or COMBO treatment was able to restore HIF- 1α activity to the levels observed during normoxia. The functional effect on HIF-1α modulation was shown by the results obtained on VEGF, a key target gene of HIF-1α (Figures 1C,D).

In order to evaluate the role of hypoxia on treatment effect, we assessed cell viability in both cell lines in absence or presence of TMZ, MET or COMBO by trypan blue exclusion test.

In U251 cells, both TMZ and COMBO significantly reduced the number of cells starting from 24 h of treatment (Figure 1E). Otherwise, in hypoxic condition, COMBO but not MET reduced viability of T98 cells starting at 48 h. To observe a significant effect of MET, 72 h needed (Figure 1F).

As regard to apoptotic genes, whereas hypoxia in U251 cells increased both pro- and anti-apoptotic genes, TMZ and COMBO promoted a balance between the up-regulation of pro-apoptotic genes, particularly Bax in COMBO and the down-regulation of the anti-apoptotic gene Bcl-2 (Figure 1G). In T98 cells, COMBO was the only treatment able to significantly increase pro-apoptotic genes and reduce the anti-apoptotic Bcl-2 gene (Figure 1H). However, the net effect was similar to that exerted by 25 μM TMZ. None of the drugs modified Caspase 3 levels in both cell lines (Supplementary Figure 1).

### MET, TMZ, and COMBO Differently Modulate Markers Associated With GBM Malignancy During Hypoxia

**Figure 2:**
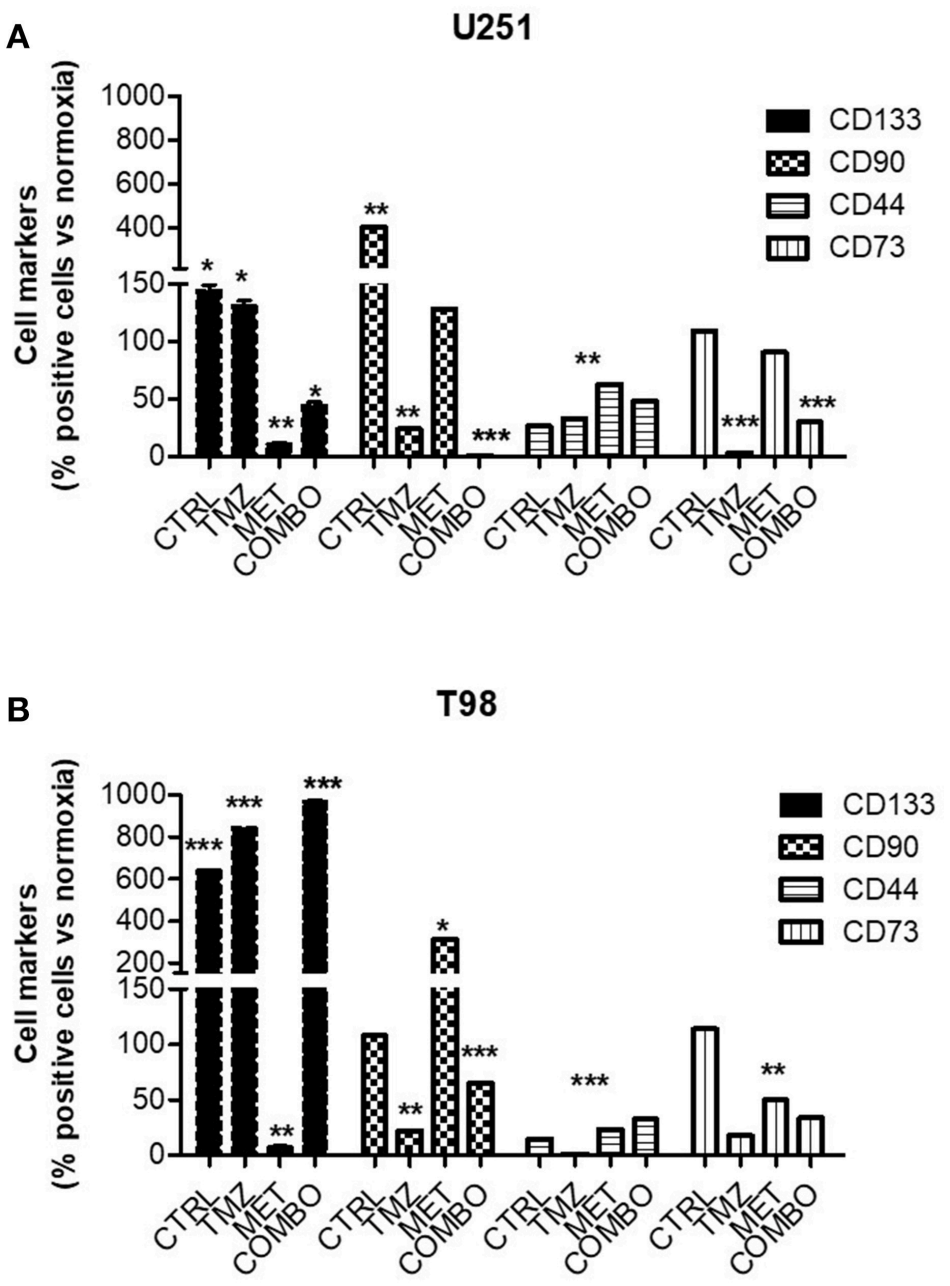
Modulation of key markers of malignancy after treatment. FACS analysis for CD133, CD90, CD44, and CD73 markers in U251 **(A)** and T98 **(B)** cells after 48 h of treatment with 25 μM TMZ and/or of 10 mM MET under hypoxia. Data were expressed as the percentage of positive cells on the number of total cells. Data are shown as the mean ± standard deviation. *p < 0.05; **p < 0.01; ***p < 0.001 vs. control sample (in normoxia).

To better understand the role of hypoxia on different cell markers of malignancy, the effect of 10 mM MET, 25 μM TMZ and COMBO on the relative abundance of CD133, CD90, CD44, and CD73 positive cells was evaluated during hypoxic condition. We previously demonstrated (12) that 10 mM MET alone and COMBO counteracted CD133 expression in U251 cells. Here, during the hypoxic condition, we observed a dramatic increase of CD133, CD90, and CD73. Only COMBO reduced the hypoxia-dependent increase in CD133, CD90, and CD73. On the contrary, when TMZ and MET were administered alone effects were marker dependent, being the first active on both CD90 and CD73 and the last only on CD133 (Figure 2A). Thus, COMBO treatment is suitable to potentiate the effects observed with TMZ treatment on all subpopulation of cells.

For what concerns T98 resistant cells, we confirmed that also in hypoxia the mechanism to overcome resistance could not be attributable to CD133 modulation. Contrary to what observed for normoxia condition, where TMZ produced an enrichment of CD90 labeled cells (12), during hypoxic condition we observed a TMZ induced reduction of this marker (Figure 2B). Despite the pronounced increase induced by MET, COMBO effects were similar to those observed for TMZ (Figure 2B). For both cell lines, we observed no or only slight effects on CD44 marker.

In addition, Real time-PCR (Supplementary Figure 2) confirmed that CD133 production was affected only in U251 in presence of MET, data that correlated with the CD133 expression on the membrane. Taken together, results on cell markers indicate that during hypoxia COMBO but not TMZ or MET alone, reduced the increase of selected cell populations, an effect that was not observed in T98 cells.

### In T98 Cells, BEZ235 Potentiates the Inhibitory Effect of COMBO on the Hypoxia-Independent Activation of HIF-1α: an Effect That Involves the PI3K/AKT/mTOR Pathway

We previously showed that TMZ dramatically reduced HIF-1α levels also during normoxic condition (18). Moreover, we observed that the effect of TMZ was similar to that obtained with BEZ235 for 48 h (18), a PI3K/AKT/mTOR modulator known to regulate HIF-1α levels in a hypoxic independent manner. In this work, we further investigated the effect of 25 μM TMZ given alone or in combination with 10 mM MET on the hypoxia-independent activation of HIF-1α levels in both responsive U251 and resistant T98 cells. Furthermore, in order to better understand the role of AKT modulation on HIF-1α regulation and treatment efficacy, drug's effects were compared with those of 1 μM BEZ235, given alone or in association with TMZ, MET or COMBO for 48 h. To this aim, a powerful strategy based on a luciferase reporter tool under the control of HRE sequences was used.

**Figure 3:**
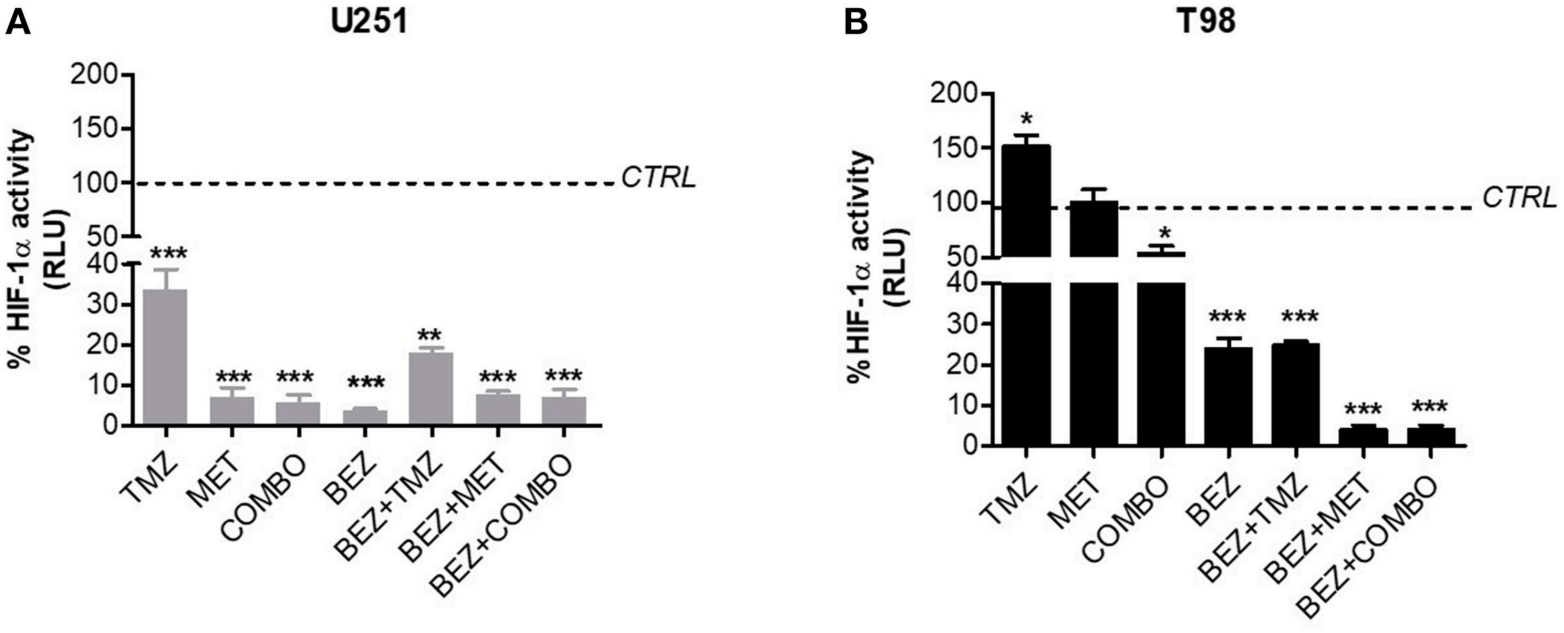
BEZ235 effect on HIF-1α activity modulation. HIF-dependent luciferase activity was analyzed in cell lysates and expressed as the percentage variation in Relative Luminescence Units (RLUs) in U251 TMZ-responsive **(A)** and T98 TMZ-resistant cells **(B)** after 25 μM TMZ, 10 mM MET, COMBO and/or 1 μM BEZ235 in combination with all the drugs for 48 h. *p < 0.05; **p < 0.01; ***p < 0.001 vs. control cells.

In U251 cells, MET, TMZ, or COMBO significantly down-regulated HIF-1α (Figure 3A). In these cells, MET, COMBO, or BEZ235 reduced HIF-1α activity, more than TMZ (Figure 3A). No add-on effect was observed when BEZ235 was associated with MET and COMBO.

In T98 cells, TMZ given alone increased HIF-1α activity. On the contrary, the association with MET reverted this effect producing a reduction of HIF-1α activity (Figure 3B). In these cells, the highest effect on HIF-1α activity was obtained when MET was associated with BEZ235 alone or plus TMZ. BEZ235 given alone or in association with TMZ reduced HIF-1α activity but to a lower extent (Figure 3B).

In order to understand if the modification on HIF-1α activity was associated with alterations in AKT activation and/or oxygenation state, we assessed the effect of TMZ, MET, and COMBO on AKT phosphorylation, during normoxic and hypoxic conditions. In the same experimental task, the effect of BEZ235 given alone or in combination was also evaluated.

**Figure 4:**
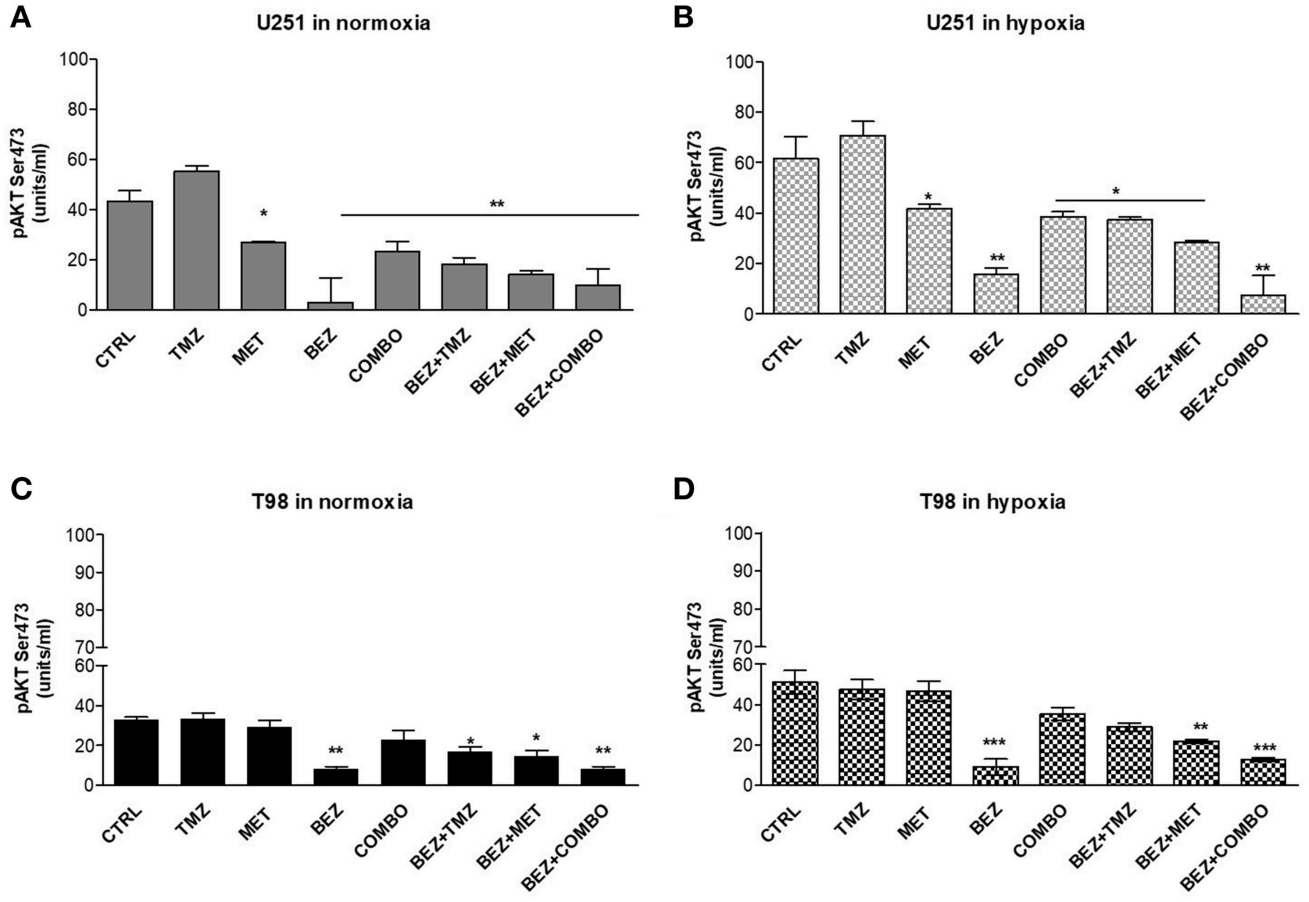
pAKT modulation by single and combined treatments. pAKT (Ser473) levels were assessed by mean ELISA assay and expressed as units/ml in normoxia **(A,C)** and hypoxia **(B,D)** in U251 **(A,B)** and T98 **(C,D)** cells after 25 μM TMZ, 10 mM MET, COMBO and/or 1 μM BEZ235 in combination with all the drugs for 48 h. *p < 0.05; **p < 0.01; ***p < 0.001 treated vs. control cells. Mean values ± SD of three independent experiments.

Cell oxygenation state only minimally influenced drug effects on AKT phosphorylation. BEZ235 given alone or in association produced the highest inhibition of AKT phosphorylation in both cell lines (Figure 4). MET or COMBO reduced AKT activation only in U251 but the reduction observed was lower than the one exerted by BEZ235 alone (Figures 4A,B). Finally, no effect on AKT activation was observed with TMZ (Figures 4A–D). These results confirm that BEZ235 effect on AKT activation is independent on hypoxia and that COMBO modulates AKT activity only in U251 cells.

### Block of PI3K/AKT/mTOR Axis Potentiates the Effect of COMBO Particularly During Hypoxic Condition

**Figure 5:**
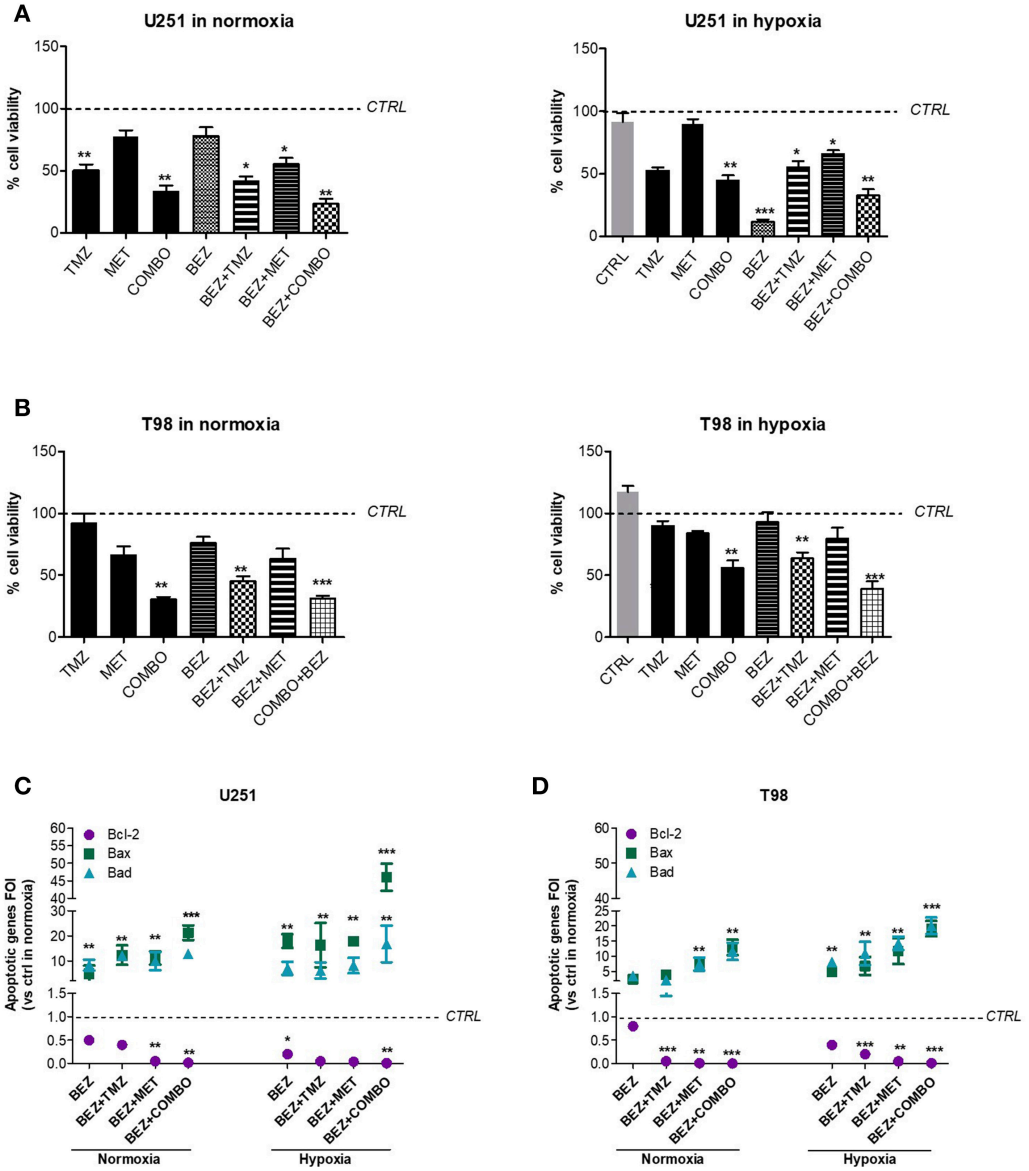
BEZ235 supports COMBO in the modulation of cell viability. Cell viability of responsive and resistant cells after 25 μM TMZ, 10 mM MET, COMBO and/or 1 μM BEZ235 in combination with all the drugs. Cell viability was assessed by means of a Trypan blue exclusion test and expressed as the percentage of viable cells after 48 h of treatment under normoxic or hypoxic conditions in U251 **(A)** and T98 **(B)** cells. *p < 0.05; **p < 0.01; ***p < 0.001 vs. control cells (i.e., untreated cells. For normoxia, control cells are represented by untreated cells in normoxia and the same normalization was used for experiments in hypoxia). The induction of pro-apoptotic (Bad and Bax) and anti-apoptotic genes (Bcl-2) was analyzed by means of real-time PCR in glioma cells treated with 25 μM TMZ, 10 mM MET, COMBO and/or 1 μM BEZ235 in combination with all the drugs for 48 h under normoxic or hypoxic conditions in U251 **(C)** and T98 **(D)** cells. The data were normalized to β-actin and the ΔΔct values were expressed as the ratio between the mean values in the responsive and resistant cells [Fold Of Induction (FOI)]. *p < 0.05; ***p < 0.001 treated vs. control cells. Mean values ± SD of three independent experiments.

After, we investigated the role of the PI3K/AKT/mTOR pathway on cell viability and apoptotic profile during normoxic and hypoxic conditions. We previously showed that hypoxia reduced the effect of 10 mM MET in T98 cells but not that of COMBO (Figure 1). In both cell lines, after 48 h treatment, 1 μM BEZ235 increased the effect of COMBO independently from the oxygenation state. BEZ235 alone reduced the viability of U251 but not that of T98 cells and only during hypoxic conditions (Figures 5A,B), suggesting that the block of AKT phosphorylation is not sufficient to reduce cell viability during the normoxic condition.

Finally, we evaluated the effect of 1 μM BEZ235 on apoptotic genes in normoxia and hypoxia. In U251 cells, BEZ235 in association with MET or COMBO promoted pro-apoptotic genes (Bax, Bad) with a significant down-regulation of anti-apoptotic Bcl-2 gene, also in hypoxic condition (Figure 5C). BEZ235 alone produced only a slight reduction in Bcl-2 during normoxia indicating that BEZ requires a reduction of oxygen to modify Bcl-2 levels. In T98 cells, BEZ235 associated with TMZ, MET or COMBO reverted the hypoxic profile restoring a “sensitive-like” pattern of apoptotic genes (Figure 5D) reaching levels higher than those observed after COMBO or TMZ alone (Figure 1H). Again, in agreement with the results obtained from cell viability, this effect was not observed when BEZ235 was given alone. Taken together these results indicate that targeting PI3K/AKT/mTOR pathway increases cell mortality during hypoxia condition also in the TMZ-resistant cell model, but only when associated with COMBO as confirmed also by the increased Caspase-3 activation in COMBO plus BEZ235- treated samples (Supplementary Figure 1). However, particularly during normoxia, the effect of BEZ235 alone is not sufficient to modify tumor cell viability.

## Discussion

In this work, first we investigated the effect of MET as a TMZ-adjuvant agent in hypoxia in GBM cell lines. In our previous paper, MET showed significant efficacy in the overcoming TMZ-resistance in a glioma model both *in vitro* during normoxic conditions and *in vivo* (12).

The aim of this study was to evaluate MET efficacy also in hypoxia, a condition that worsens cell phenotype. This involves hypoxia-inducible factors, stem population, negative modulation of apoptotic axis and molecular pathways related to the regulation of cell metabolism including PI3K/AKT/mTOR pathway.

One of the anticancer effects of MET is based on modifications of the balance between AMPK activation and mTOR inhibition, which leads to the negative modulation of HIF-1α (20). Moreover, in a previous study, we showed that a significant reduction in HIF-1α activity preceded a response to TMZ treatment in GBM cells (18), a fact that led us to further explore this effect also during combined treatments.

Here, we observed that hypoxia worsened cell phenotype, increasing HIF-1α levels, anti-apoptotic Bcl-2, and markers of malignancy, like CD133 and CD90, this last particularly in T98.

We first studied the effect of single and combined treatment on HIF-1α activity in GBM cells. Our results showed that COMBO was able to reduce HIF-1α activity not only in U251-responsive cells but also in T98 TMZ-resistant cells. Both MET and TMZ act on AMPK (9) thus modulating HIF-1α activity and, as shown by cell viability, COMBO administration reverted the resistant-like pattern of T98, also in hypoxia (Figure 1). However, differently to what observed in U251, in T98, the increased efficacy of COMBO in comparison to TMZ cannot be related to the modulation of apoptotic genes.

Among other processes that can be associated to an AMPK-dependent positive modulation of Forkhead box O3 (FOXO3) and a negative modulation of mTOR/AKT (13), MET may specifically act on tumor-initiating GSC cells. However, a recent study showed that in tumors with constitutive activation of AMPK, the anti-proliferative effect of MET on GSCs is probably independent of AMPK (21).

Since hypoxia and HIF-1α are known to be involved in promoting the self-renewal capacity of CD133 (22) -and CD90 positive cells, to understand the effect of MET on glioma markers of malignancy, U251 and T98 cells were analyzed in hypoxic condition for the expression of these markers together with CD73 and CD44. Herein we demonstrated that COMBO was able to counteract the hypoxia-induced increase of CD133, CD90, and CD73 in TMZ-responsive cells but only that of CD90 and CD73 markers in TMZ-resistant cells, confirming the results obtained previously in normoxia (12). However, during hypoxic condition, the effect of COMBO was close to that of TMZ. This result provides evidence that, during hypoxia, MET potentiates the effect of TMZ only in U251 cells (Figure 2).

Furthermore, it has been recently discussed that MET was able to block proliferation on glioma tumor-initiating cells through the inhibition of the AKT-mTOR pathway (21). It has been also described that AKT can be activated by TMZ and the overexpression of its active form is positively correlated to GBM cell resistance to TMZ. Conversely, the block of AKT-mTOR pathway promotes TMZ responsiveness (23). AKT-mTOR block is of particular interest in GBM since more than 40% of lesions shows an activated pathway thanks to the loss of function or reduced expression of the PTEN gene. Recent evidence suggested that AMPK and AKT display antagonistic roles under metabolic stress (24), such as in a hypoxic condition.

In addition to their mutual regulation based on specific phosphorylation, AMPK and AKT have at least two common targets: mTOR and FOXO. Indeed, whereas AMPK blocks mTOR and activates FOXO, AKT is involved on the contrary process. Thus, MET, by activating AMPK, inactivates AKT through its dephosphorylation at Ser-473 and Thr-308 thus impairing HIF-1α activity (24).

For these reasons, the effect of the block of AKT activation has been herein explored by using a dual PI3K/AKT and mTOR inhibitor, BEZ235 given alone or in combination with AMPK modulators. The anticancer effect of this drug has been evaluated in preclinical studies (25, 26), and recently in phase 1 clinical trial for advanced solid tumors (colorectal, breast, non-small cell lung carcinoma, renal, and sarcoma) (27). Dual PI3K-mTOR inhibitors are particularly effective in blocking AKT activation because they prevent the feedback activation of PI3K signaling normally observed with mTORC1 inhibitors (28).

We firstly investigated the effect of BEZ235, MET, and TMZ on hypoxia independent HIF-1α modulation when given alone or in combination. Then, we studied the specific involvement of the PI3K/AKT pathway by analyzing the phosphorylation form of AKT in normoxia and hypoxia. TMZ, MET, and COMBO reduced HIF-1α but only in U251 cells. In T98, only COMBO was able to reduce HIF-1α although its effect was modest, whereas TMZ administration increased its levels (Figure 4). As expected, BEZ235 significantly reduced HIF-1α activity both in TMZ-sensitive and -resistant cells, with a stronger effect when combined with MET or COMBO and prevented the increase induced by TMZ in T98 (Figures 3A,B). Partially in line with these data, TMZ failed to reduce AKT phosphorylation independently from cell line or condition. MET and COMBO displayed some effects but only in TMZ responsive U251 cells. BEZ235 given alone or in combination with MET or COMBO displayed the highest inhibition of AKT phosphorylation.

Hypoxia-independent inhibition of HIF-1α and AKT phosphorylation exerted by BEZ235 were not associated with the modification of the viability of both cell lines. On the contrary, PI3K/mTOR inhibition reduced cell viability of U251 but not that of T98 during hypoxic condition. These results are in line with the apoptotic profile: BEZ235 was able to reduce Bcl-2 level only during hypoxic condition and in U251 cells. This last effect is reasonability related to the block of the hypoxia-dependent activation of AKT pathway or to a direct effect on mTOR. Interestingly, a recent study showed that the inhibition of hypoxia-dependent mitochondrial AKT activation pathway was followed by an increase of cell apoptosis and reduction of cell viability (29). This is not the case of T98 cells, where BEZ235 was not able to produce any effect on cell viability and apoptosis also during hypoxic conditions. Indeed, in TMZ resistant cells, reduction of cell viability was observed only after COMBO administered alone or combined to BEZ235 (Figure 5B). On the other hand, in this cell line, BEZ235 associated with MET, TMZ, and particularly with COMBO promoted a significant down-regulation of Bcl-2 anti-apoptotic gene and an up-regulation of Bax and Bad genes, independently from oxygenation state. These results suggested that the cooperation between the drugs could help the cells to modulate the apoptotic switch through a double targeting of mTOR (AMPK and PI3K/mTOR) resulting finally in the reduction of cell viability and in the negative modulation of HIF-1α activity.

Another potential player of MET activity is JNK/p38 MAPK, a stress-activated kinase involved in the regulation of apoptosis (30). Indeed, it has been recently suggested that p38 is a direct target of MET, involved in the activation of p53 pathway and finally in the Bax-mediated promotion of apoptosis (31). Moreover, modulation of p38 pathway modifies the sensitivity of cells to methylating agents, such as TMZ (32) (O6mG lesions and their processing by the MMR system were critical for p38α activation).

In conclusion, results obtained herein suggest that MET is able to revert TMZ-resistance also in hypoxia, and its effect involves the modulation of HIF-1α activity, at least in *in vitro* settings. In U251 TMZ-responsive cells, the association with MET increases the effect on markers of malignancy further addressing the relevance of COMBO. Finally, the promotion of a pro-apoptotic phenotype induced by BEZ235 when is added to COMBO in T98 cells, set the basis to further investigate *in vivo*, in preclinical orthotopic models, a triple therapy approach based on the administration of COMBO plus double PI3K/mTOR inhibitor.

## Author Contributions

AL and SV: data generation, collection, analysis and interpretation, and manuscript drafting. LO and RM: study conception and design, and critical review of the manuscript. All the authors approved the final version of the manuscript.

### Conflict of Interest Statement

The authors declare that the research was conducted in the absence of any commercial or financial relationships that could be construed as a potential conflict of interest.
