# Supplementary figures and images for: Role of Metformin and AKT Axis Modulation in the Reversion of Hypoxia Induced TMZ-Resistance in Glioma Cells
Source: Front Oncol. 2019 May 31;9:463. doi: 10.3389/fonc.2019.00463 (PMC6554426; doi:10.3389/fonc.2019.00463)

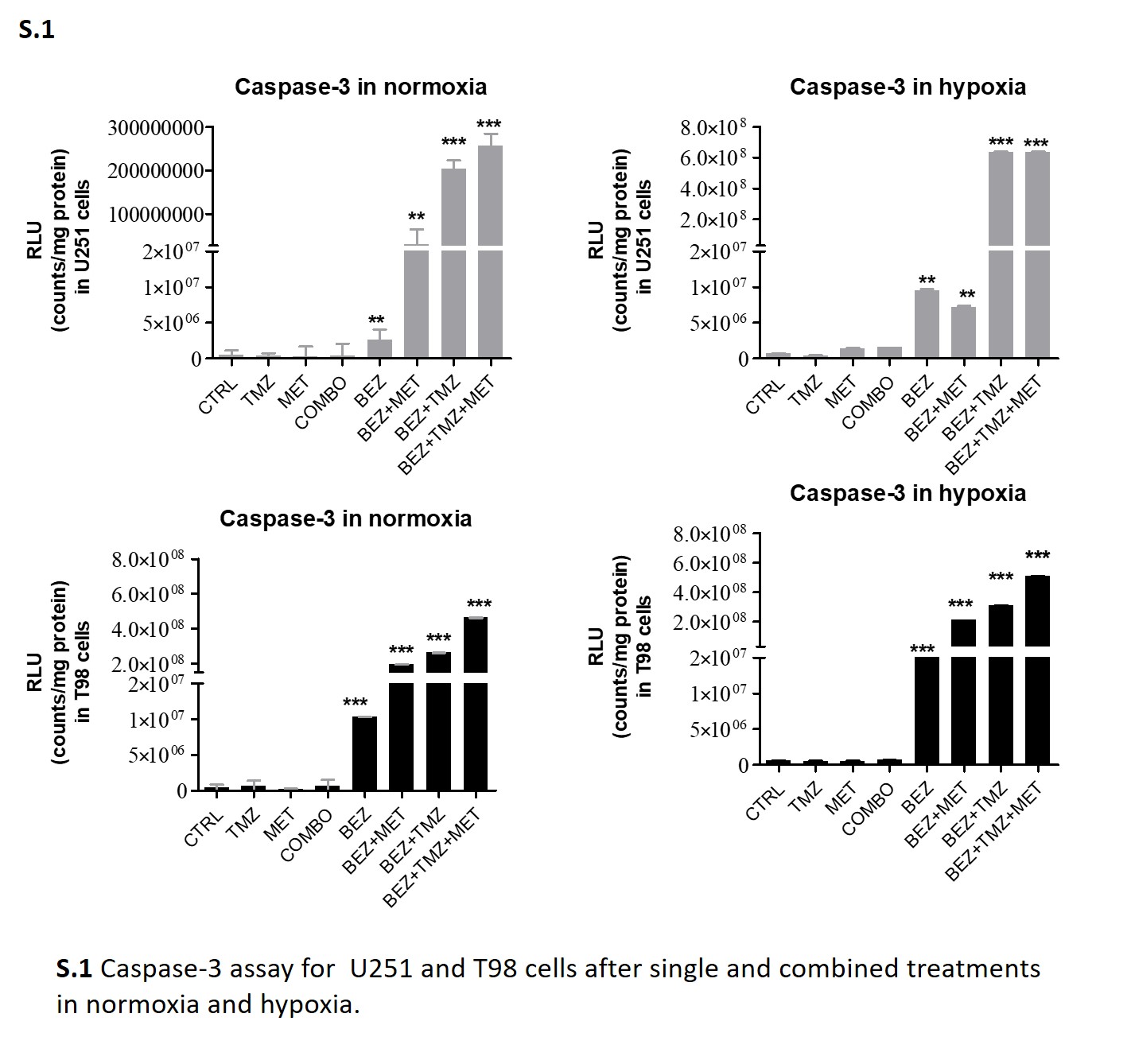

Supplement: Supplementary file 1 [file Image_1.JPEG]

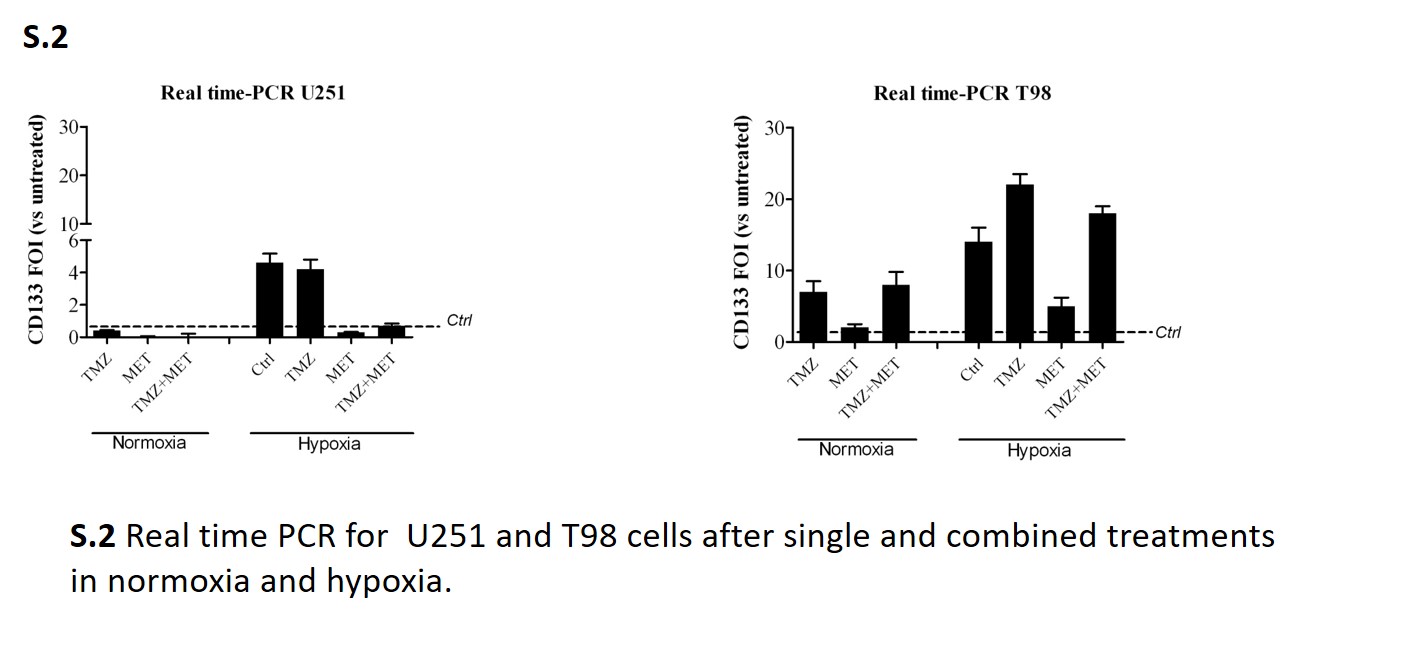

Supplement: Supplementary file 2 [file Image_2.JPEG]
